# Phytosterol Profiling as a Tool for Edible Oil Authentication: Challenges and Prospects

**DOI:** 10.3390/foods15061101

**Published:** 2026-03-20

**Authors:** Kaili Cheng, Tong Zhou, Wei Wang, Jiuliang Zhang, Xiaoting Zhou, Bing Hu, Tao Zhang

**Affiliations:** 1 College of Food Science and Technology, Huazhong Agricultural University, Wuhan 430070, China; catt@webmail.hzau.edu.cn (K.C.); zzz111@webmail.hzau.edu.cn (T.Z.); zjl_ljz@mail.hzau.edu.cn (J.Z.); 2 Key Laboratory of Edible Oil Quality and Safety, State Administration for Market Regulation, Wuhan 430012, China; wangwei881215@sina.com (W.W.); hbydycf@163.com (B.H.)

**Keywords:** phytosterols, edible oils, chemometrics, sterol profiling, adulteration

## Abstract

The global edible oil market is consistently at risk of economically motivated adulteration, underscoring the necessity of robust analytical methods essential for authentication. Among various phytochemicals, phytosterols have emerged as powerful diagnostic markers and compositional indicators for verifying the botanical origin, purity, and quality of edible oils. This review summarizes recent advancements in phytosterol analysis, highlighting its application in detecting adulteration in high-value oils such as olive oil, tea seed oil, and sesame oil. We discuss the approaches of multiple chromatographic and mass spectrometry techniques (GC-MS, LC-MS) with chemometric analysis of novel markers like fatty acyl sterol esters and sterol degradation products. Furthermore, we discuss significant challenges, including the need for comprehensive databases, the identification of complex sterol compositional profiles, and the limitations of current standardized methods. The advancement of phytosterol-based authentication increasingly depends on the development of rapid, high-throughput, and non-targeted sterol profiling approaches, supported by artificial intelligence and bioinformatics, to ensure vegetable oil authenticity and safeguard market integrity.

## 1. Introduction

Edible oil authentication represents a critical frontier in food safety, driven by the growing challenge of economically motivated adulteration. The high market value of premium oils, such as olive oil, Camellia seed oil, and sesame oil, creates a powerful incentive for their fraudulent dilution with cheaper oils or inferior alternatives [1]. Conventional methodologies for verifying oil quality have relied on assessing basic physicochemical parameters such as acidity, peroxide value, or fatty acid composition. A critical weakness of these approaches, however, is their susceptibility to strategically formulated fraudulent blends that replicate key chemical characteristics of the authentic oil, creating a significant analytical challenge for targeted authentication [2]. Thus, edible oil authentication is in urgent demand of more specific and reliable chemical markers and compositional features that can serve as robust evidence of origin and purity.

**Figure 1:**
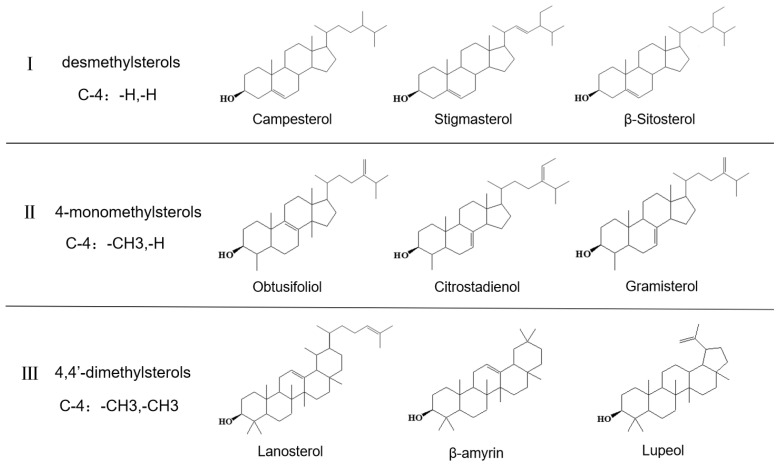
Schematic illustration of the structural basis underlying phytosterol profiling in edible oils. The common steroid nucleus is shown as a shared framework, while key diagnostic variations relevant to authentication are highlighted, including the degree of C4 methylation, side-chain substitution at the C24 position, and the position of double bonds within the sterol structure.

Phytosterols, a class of plant-derived sterols, have emerged as crucial biomarkers for authenticity verification. As integral components of the unsaponifiable fraction of edible oils, phytosterols are not hydrolyzed during standard oil processing and remain as a stable chemical signature [3]. Their compositional profiles are highly specific to botanical origin, as distinct biosynthetic pathways in different plant species produce characteristic sterol distributions [4]. This inherent specificity renders phytosterol compositional profiles highly characteristic of individual oil types, making them more difficult for adulterators to replicate accurately than simple fatty acid compositions [5,6]. The phytosterol family exhibits remarkable structural diversity, encompassing three major classes, including desmethylsterols (β-sitosterol, campesterol, stigmasterol), 4-monomethylsterols, and 4,4′-dimethylsterols (triterpene sterols) [7], with representative structural features illustrated in Figure 1. Beyond their analytical utility, certain minor sterol subclasses such as 4,4-dimethylsterols may also have nutritional relevance, further increasing the value of comprehensive sterol profiling [7,8]. Each sterol class contributes to a multi-dimensional compositional dataset that can be exploited for fine-scale discrimination between closely related oils [4].

**Figure 2:**
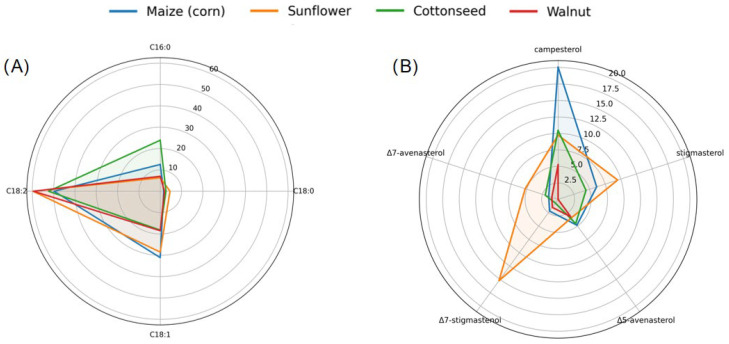
Schematic comparison of fatty acid composition and phytosterol compositional profiles in representative edible oils. (**A**) Radar plot of major fatty acids (C16:0, C18:0, C18:1, and C18:2; % of total fatty acids) for maize (corn), sunflower, cottonseed, and walnut oils. (**B**) Radar plot of phytosterol compositional profiles (campesterol, stigmasterol, Δ5-avenasterol, Δ7-stigmastenol, and Δ7-avenasterol; % of total sterols) for the same oils.

In food authentication studies, marker-based, profile-based, and fingerprint-based methodologies represent conceptually distinct analytical strategies. Marker-based approaches rely on one or a few well-defined compounds characteristic of a product or adulterant, whereas profile-based approaches exploit the compositional patterns and relative distributions of multiple identified compounds. In contrast, fingerprint-based authentication is a non-targeted strategy that uses holistic analytical signals without prior compound identification. In phytosterol research, current applications in edible oil authentication predominantly employ marker-based and profile-based approaches, while non-targeted sterol profiling represents an emerging direction. Practically, phytosterol profiling leverages oil-specific sterol compositional patterns (and, where useful, simple diagnostic sterol ratios) to discriminate oils, providing complementary contrast in cases where several oils show overlapping fatty acid compositions but differ in sterol composition, with an illustrative comparison shown in Figure 2.

The development of advanced analytical technologies for plant sterols, particularly high-resolution chromatographic and mass spectrometry techniques, has dramatically expanded the scope of sterol analysis. For example, earlier studies focused on a limited panel of a few desmethylsterols, while the latest research has enabled the comprehensive profiling of over 150 different sterols and related triterpenoid compounds [9], uncovering a previously hidden layer of phytosterol diversity.

**Figure 3:**
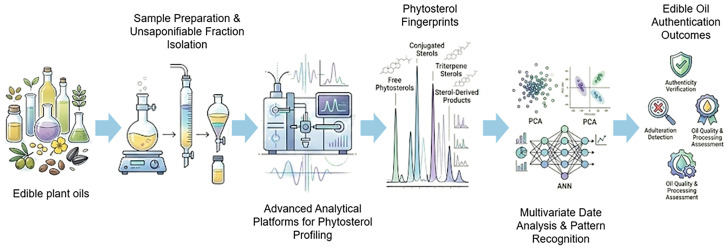
General workflow of phytosterol profiling for edible oil authentication. The figure outlines the key steps involved in phytosterol analysis, including sample preparation, separation, and detection of specific sterol markers, together with the major analytical techniques applied to generate sterol profiles for the authentication of edible oils.

In light of these advancements, this review critically evaluates phytosterol-based markers and compositional profiling for edible oil authentication. Compared with prior broad authentication reviews, our contribution is a phytosterol-centered, decision-oriented synthesis that links oil type, likely adulterant scenarios, sterol markers/profiles, analytical methods, and validation considerations into a unified framework, as shown in Figure 3. Finally, the review will provide a forward-looking perspective on integrating this approach into robust authenticity frameworks for edible oil.

## 2. Analytical Techniques for Phytosterol Profiling

### 2.1. Sample Preparation and Separation

Efficient and selective sample preparation is critical for accurate phytosterol analysis, as it directly influences the resolution, sensitivity, and reliability of the phytosterol compositional profiling. This shift from traditional, labor-intensive techniques such as thin-layer chromatography (TLC) toward automated, high-throughput methodologies marks a significant advancement in phytosterol profiling. In particular, solid-phase extraction (SPE) has emerged as a superior alternative for the purification and fractionation of complex phytosterol classes from the unsaponifiable matter of vegetable oils [10]. Previously, Azadmard-Damirchi and Dutta developed a SPE protocol utilizing a stepwise elution with n-hexane and diethyl ether [11]. This method achieved effective separation of 4-desmethyl-, 4-monomethyl-, and 4,4′-dimethylsterols, with higher recovery rates and greater reproducibility than conventional TLC. Complementing this approach, a sophisticated two-step SPE procedure was established for olive oil analysis, employing an initial polymeric sorbent followed by a silica gel cartridge [12]. This technique enabled rapid and simultaneous extraction of 4-desmethylsterols and triterpene sterols, showing excellent agreement with official reference methods while offering enhanced speed and reduced solvent consumption. Recently, magnetic solid-phase extraction (MSPE) has emerged as a modern alternative to conventional SPE, primarily due to its innovative use of magnetic separation. For example, the recovery of β-sitosterol reached 86% using a magnetic graphene oxide nanocomposite, representing a significant enhancement over traditional adsorbents [13]. Engineered β-sitosterol-imprinted magnetic beads (mag-MIP) exhibit superior selectivity and capacity compared to non-imprinted counterparts (mag-NIP), yielding high recoveries and substantial enrichment factors for trace analysis [14]. Thus, the MSPE approach bypasses the need for specialized cartridges and centrifugation, leading to faster processing times without compromising extraction performance.

### 2.2. Gas Chromatography-Based Methods

**Figure 4:**
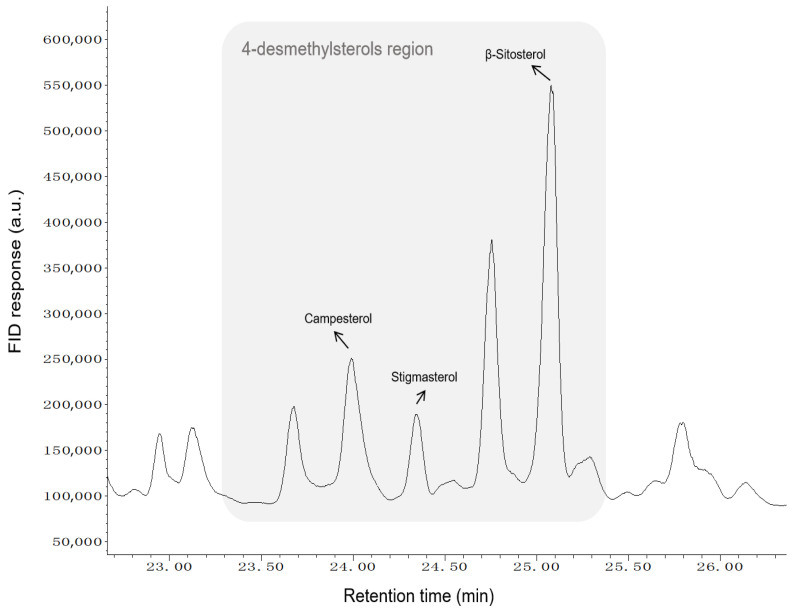
Representative GC–FID chromatogram of derivatized phytosterols from rice bran oil. The shaded area indicates the 4-desmethylsterols region; the major peaks (campesterol, stigmasterol, and β-sitosterol) are annotated.

Gas chromatography (GC) stands as a cornerstone technique for phytosterol analysis, enabling both qualitative and quantitative assessment. Its efficacy stems from the use of capillary columns (e.g., HP-5, DB-5) [15], which generally provide higher resolution than a high-performance liquid chromatography (HPLC) column (C18). When sample complexity challenges one-dimensional gas chromatography (1D-GC), two-dimensional gas chromatography (2D-GC) provides enhanced separation by expanding the dynamic range and mitigating matrix effects [16]. Detection is most commonly achieved using mass spectrometry (MS), which combines high-efficiency separation with the strong qualitative capability, or flame ionization detection (FID), which is prized for its sensitivity and wide linear range. Advances in MS technology, including triple quadrupole (QQQ) and time-of-flight (TOF) instruments, continue to push the boundaries of detection sensitivity [17,18]. However, a critical sample preparation step for GC analysis is derivatization. The conversion of phytosterols to their TMS ethers is a well-established method that ensures volatility and thermal stability, leading to excellent chromatographic results and high recovery rates. Accordingly, as an illustrative example, a GC–FID chromatogram of derivatized phytosterols from rice bran oil is shown in Figure 4, highlighting the 4-desmethylsterols region and the major peaks used for compositional profiling.

The accurate determination of phytosterol compositional profiles is critical for edible oil authentication [19]. GC-FID is the basis for sterol analysis in many international official standard methods [20]. However, these methods are often criticized for being time-consuming, labor-intensive, and requiring large amounts of solvents [12]. Recent innovations focus on automation and miniaturization. Nestola and Schmidt developed a fully automated online LC-GC-FID method that integrates saponification, extraction, and analysis, achieving a sample throughput of one sample per hour [21]. Furthermore, this method successfully identified an additional sterol, 14-methyl fecosterol, in sunflower oil that was previously masked by derivatization in the official methods. GC-MS, particularly in selected ion monitoring (SIM) mode, offers superior sensitivity and specificity. Recently, Schlag et al. utilized GC-MS-SIM and created a seminal database detailing the occurrence and semi-quantitative levels of 150 sterols in 74 different vegetable oils and other matrices [9]. This database represents an important resource for identifying unusual sterols that may serve as diagnostic markers for specific edible oils.

### 2.3. Liquid Chromatography–Mass Spectrometry (LC-MS)

While GC methods often require derivatization to increase volatility, LC-MS allows for the analysis of intact sterols and their esters [22]. Developing a robust HPLC method for phytosterol analysis requires optimization of several chromatographic parameters, such as the stationary and mobile phases, along with various additives. Reversed-phase columns, such as C18 and phenyl stationary phases, are frequently employed for phytosterol separation. The C18 column is widely recognized for its effective separation of phytosterols. Using a Zorbax Eclipse Plus C18 column, phytosterols, tocopherols, and squalene in vegetable oil distillates were effectively separated within 33 min [23]. The phenyl column offers unique selectivity due to the presence of a benzene ring in its stationary phase, enabling π-π interactions with analytes that enhance retention and separation. This advantage was confirmed by a previous study, in which a phenyl column was selected over C8, C18, and C30 phases for the simultaneous analysis of phytosterols, tocopherols, and lutein in soybeans due to its superior performance [24].

Advances in liquid chromatography include ultra-high performance liquid chromatography (UHPLC), which utilizes smaller particle sizes (1.7–3 µm) to deliver higher resolution, improved accuracy, and significantly reduced run times. The UHPLC BEH C18 column enables the separation of six phytosterols in tobacco within only 8 min, while concurrently reducing solvent consumption [25]. Similarly, another study completed the analysis of tobacco leaf phytosterols within an even shorter time (6 min), underscoring that the UHPLC approach aligns with the principles of green chemistry [26]. Another advancement is capillary HPLC (cap-HPLC); owing to its narrower inner diameter, it provides higher separation efficiency, faster analysis, and minimal consumption of samples and solvents. For example, using a capillary C18 column, polyphenols and phytosterols from grape seeds were effectively separated, achieving impressively low detection limits for stigmasterol [27].

The composition of the mobile phase is also critical for optimizing analytical time and peak morphology. Given their hydrophobic character, phytosterols are strongly retained on reversed-phase columns, often necessitating a highly organic mobile phase. While mixtures of methanol or acetonitrile with water are widely used, solvent systems such as hexane and 2-propanol are also employed [28,29]. To further improve chromatographic performance, additives including ammonium acetate, formic acid, acetic acid, trifluoroacetic acid, and KH_2_PO_4_ are commonly incorporated. With respect to LC-MS methods, a previous study developed a non-aqueous reversed-phase LC-MS approach for simultaneous analysis of free phytosterols and phytosterol esters bearing various fatty acids [30]. This approach could be readily adapted for edible oil analysis, providing a more detailed phytosterol compositional profile without the need for prior hydrolysis. In addition, LC-MS methods have been applied to human serum for the detection of dietary phytosterols, demonstrating their sensitivity and applicability to complex biological matrices [31].

From a practical perspective, high-resolution platforms (e.g., UHPLC–QTOF/HRMS) are particularly advantageous when comprehensive profiling and structural elucidation are needed, but they generally demand greater instrument investment and more specialized operation. By contrast, LC–MS/MS approaches are often favored for targeted panels once the method is established, because they provide robust quantitation with efficient sample-to-result workflows. GC-based methods remain highly useful where standardized sterol panels and inter-laboratory comparability are priorities, although sample preparation requirements can become the rate-limiting step for throughput. Emerging rapid-screening approaches can further increase throughput for large-scale surveillance, but typically benefit from confirmation by GC–MS or LC–MS when unambiguous identification is required. A comparative overview of the analytical techniques discussed in this section is presented in Table 1.

### 2.4. Data Processing and Chemometric/Machine Learning Tools for Sterol-Based Authentication

Sterol-based authentication is inherently multivariate; therefore, the reliability of classification depends critically on data processing and chemometric analysis. Phytosterol profiling data are typically organized as a samples × variables matrix using absolute concentrations, relative compositions (% total sterols), and/or diagnostic ratios, and may be integrated with complementary chemical classes such as fatty acids to enhance discrimination in complex scenarios [35,36]. Routine preprocessing generally includes normalization (e.g., internal standard, sample amount, total sterols or unit-sum for compositional profiles), mean-centering, and scaling (often autoscaling or Pareto scaling), together with explicit handling of values below LOD/LOQ and missing data; for multi-batch datasets, QC monitoring and drift/batch correction can be applied, provided that preprocessing parameters are derived within the training set to avoid information leakage.

Commonly used tools include unsupervised methods (e.g., PCA and HCA) for exploratory assessment and supervised models for authentication tasks. In practice, supervised multivariate analysis (PLS-DA/OPLS-DA) and machine learning classifiers such as Random Forest, SVM, and artificial neural networks (ANN) are frequently used; for example, combined untargeted profiling of phenolics and sterols with OPLS-DA and ANN enabled geographical origin and variety discrimination of EVOO with high sensitivity [36]. Variable selection/interpretation is often supported by metrics such as VIP from OPLS-DA, which has been used to prioritize informative sterol-related ratios (e.g., linoleic acid/stigmasterol) and improve adulterant-type discrimination [37]. Because modern profiling generates high-dimensional data, chemometric analysis is increasingly necessary for interpretation [36,38]; however, authentication studies must explicitly address common pitfalls, including overfitting in small-sample settings, data leakage from improper preprocessing before splitting, and performance inflation when external validation is absent. Accordingly, studies should transparently report preprocessing steps, model type and key settings, validation design (train/test split and/or cross-validation strategy), and robust performance metrics (confusion matrix with class-wise sensitivity/specificity; balanced metrics when classes are imbalanced), together with chemically plausible interpretation of discriminant sterol features.

## 3. Applications in Edible Oil Authentication

### 3.1. Olive Oil

The authentication of virgin olive oil and the detection of its adulteration with lower-priced oils remain significant challenges in the olive oil market. Phytosterols serve as critical chemical markers for assessing the authenticity and purity of olive oil. Their distinct compositional profiles facilitate discrimination of olive oil from other vegetable oils and enable detection of adulteration with lower-priced products, such as the addition of solvent-extracted olive oil to extra virgin olive oil (EVOO). Specific sterol ratios are well-established indicators of adulteration. For instance, campesterol and stigmasterol ratios can reveal the presence of corn, soybean, sunflower, and cottonseed oils [39,40], while uvaol and erythrodiol are diagnostic markers for solvent-extracted olive oil [40]. Other phytosterols, including Δ7-stigmastenol and campesterol, can serve as effective markers for sunflower and corn oil adulteration [41], and both have also been validated for detecting soybean oil [42]. Comprehensive analytical panels combining total sterol content, desmethylsterol composition, and triterpene sterols have successfully identified adulteration with several common vegetable oils, although this approach may fail to detect hazelnut oil [43]. Lupeol and an additional lupane-type compound (compound X) have been reported to be detected only in the 4,4′-dimethylsterols fraction of hazelnut oil, accounting for approximately 6–10% and 2–8% of this fraction, respectively. Under the reported conditions, the presence of these markers enabled the detection of hazelnut oil adulteration in virgin olive oil even at admixture levels below 4% [44]. Recent methodological advances have further enhanced analytical sensitivity. By analyzing both free and esterified sterols alongside triterpenic alcohols, adulteration with as little as 2% hazelnut oil and 2% sunflower oil was successfully detected [45,46]. Beyond authenticity assessment, sterol compositional profiles can discriminate EVOOs according to cultivar origin. For example, Δ5-avenasterol content can range from 2.2% to 15.2% of the total sterol fraction [47].

To further improve detection capabilities, sterol profiling is increasingly combined with complementary analytical platforms and multivariate data analysis. For example, EVOOs have been classified by ^1^H NMR spectroscopy using lipid signal suppression to enhance sterol resonances and other diagnostic features [48]. Multivariate models based on combined sterol and fatty acid datasets have differentiated EVOOs adulterated with cottonseed and sunflower oils [35]. Moreover, combined untargeted profiling of phenolics and sterols enabled the identification of the geographical origin and variety of Taggiasca Ligure EVOOs, with a sensitivity of 100% as reported in the authors’ dataset and validation setting [36]. Refined olive oil adulteration has also been assessed using joint phenolic and sterol information [49]. Compared with traditional approaches, this strategy was reported to improve identification performance under the experimental conditions described in that study [45].

The diagnostic power of sterols can be further complemented by stigmastadiene analysis, as these compounds are highly sensitive markers for refined oil adulteration. While virgin olive oils from cold extraction contain negligible amounts of stigmastadienes (<0.01 mg/kg), regulatory limits for EVOO are set below 0.15 mg/kg [50]. These compounds are formed during the refining process, where high temperatures induce the dehydration of sterols such as β-sitosterol, leading to measurable concentrations of 3,5-stigmastadiene (0.3–0.9 mg/kg) [51].

### 3.2. Camellia Seed Oil

The traditional cornerstone for camellia seed oil (CSO) authentication has been the quantification of fatty acid composition, particularly its high oleic acid content, which typically exceeds 80% of total fatty acids. However, the emergence and cultivation of other “high-oleic” vegetable oils (e.g., high-oleic sunflower and high-oleic safflower oils) has created a significant analytical challenge. These oils can exhibit oleic acid concentrations that overlap with CSO, rendering fatty acid composition alone an unreliable marker for authentication. This limitation necessitates the exploration of more sophisticated chemical markers unique to the Camellia oleifera species, moving beyond bulk compositional analysis toward the identification of specific sterol components that serve as diagnostic markers.

Recent research has demonstrated that CSO is characterized by a uniquely high concentration of triterpene 4,4′-dimethylsterols, particularly β-amyrin and lanosterol, which are negligible or markedly lower in most common vegetable oils. Accordingly, multivariate evaluation of sterol compositional profiles shows that CSO clusters distinctly from other oils, primarily driven by high loadings of β-amyrin and lanosterol, supporting their use as characteristic markers for CSO authentication [5,52]. Therefore, these two compounds can be established as characteristic markers, providing a robust chemical basis for the authentication of pure camellia seed oil. However, beyond distinguishing CSO from common vegetable oils like soybean or rapeseed oil, sterol analysis can also address more challenging forms of adulteration. For instance, β-sitosterol has been identified as a potential marker for differentiating between virgin CSO and virgin olive oil with similar fatty acid profiles. Studies indicate that the concentration of β-sitosterol in virgin CSO falls within 14.1–30.2 mg/100 g, whereas in virgin olive oil it is substantially higher (94.3–173.2 mg/100 g) [53]. This clear disparity provides a quantifiable metric to detect adulteration between these two high-value oils.

To facilitate application, CSO adulteration can be categorized into three primary types. The first involves adulteration with common vegetable oils (e.g., corn, soybean, or canola oil); this type can often be screened by deviations in bulk composition and supported by changes in characteristic sterols (e.g., β-amyrin and lanosterol), although systematic validation across diverse adulterants and blending ratios remains limited. The second involves adulteration with other high-oleic oils; this form is problematic because it bypasses the oleic acid criterion, and the sterol compositional profiles may be less distinct, demanding more specific markers or advanced analytical techniques. The third involves blending high-grade CSO with low-grade, often refined CSO; this is difficult to detect because the core chemical constituents are similar, requiring detection of subtle changes in minor components or physicochemical properties altered during refining.

In response to these challenges, three-dimensional fluorescence spectroscopy has been explored as a rapid, non-destructive screening approach for CSO adulteration, leveraging native fluorophore signals in the oil matrix to discriminate pure and adulterated samples [54]. This provides a high-throughput complement to targeted sterol profiling for confirmatory analysis.

### 3.3. Sesame Oil

Sesame oil, a valuable commodity derived from sesame seeds, is prized for its characteristic flavor and nutritional profile. In China, its high market price, 3~10 times greater than that of common edible oils, has led to a high incidence of adulteration. Typically, cheaper oils such as soybean, palm, and cottonseed oils are used as adulterants. This practice not only violates fundamental consumer rights, including the right to accurate information and fair value, but also poses a serious threat to public health.

Chen et al. screened suspicious samples using combined information from fatty acids, tocopherols, and phytosterols, and subsequently confirmed rapeseed oil adulteration using brassicasterol as a specific marker [38]. In a related study, sterol profiling was used to trace the adulteration of sesame and niger seed oils with palm oil [55]. In all oils analyzed, sitosterol, campesterol, and stigmasterol were the dominant sterols. Lupeol, lanosterol, and olean-12-en-3-one were found in significant proportions exclusively in niger seed oil, whereas cholesterol and 24-nor-22,23-methylenecholest-5-en-3β-ol served as unique identifiers for palm oil. This study detected palm oil adulteration at levels as 10% in both niger seed and sesame oils by tracing these unique sterol markers.

### 3.4. Other Oils

For other oils, Pumpkin seed oil is characterized by an unusual sterol pattern rich in Δ7-sterols, which contrasts with most common seed oils where Δ5-sterols typically predominate. Owing to its relatively high market value, pumpkin seed oil is occasionally adulterated with cheaper refined oils (often sunflower oil), and sterol compositional profiling has been used as a confirmatory tool for authenticity assessment in retail samples [56]. Rodríguez-Sánchez et al. [2] emphasized that although phytosterol compositional profiles can be replicated, unique fatty acyl esters found in *Persea* species, such as Persin and Persenone A, serve as irreplaceable chemotaxonomic markers for authenticating pure avocado oil. Beyond specialty oils, adulteration is a concern in fat-containing dairy products. For instance, Dowlatabadi et al. employed a dual-strategy combining qPCR with GC-FID-based sterol analysis to detect and quantify palm oil adulteration in yogurt, reporting a strong correlation between the two methodologies [57].

The specific composition of sterols and their degradation products provides critical evidence of adulteration and processing history. Zhao et al. demonstrated that the contents and ratios of cholesterol, β-sitosterol, and campesterol could be used to identify vegetable oils adulterated with waste cooking oil [58]. The presence of cholesterol, which is atypical in refined vegetable oils, alongside altered sterol ratios, served as key indicators. Similarly, stigmasta-3,5-diene, formed from sterols during refining and high-temperature deodorization, acts as a definitive marker for the presence of deodorized or adulterated oils [59,60].

Beyond detecting simple adulteration, sterol compositional profiles can also trace geographic origin, enabling the differentiation of samples originating from sites only ~250 km apart in the investigated dataset [61]. Creydt and Fischer applied non-targeted metabolomics to spruce wood and demonstrated that non-polar metabolites, including phytosterol, can be used for origin differentiation—a concept directly applicable to edible oils, where environmental factors distinctly influence metabolic profiles and enable origin verification [36,62]. An overview of characteristic phytosterol markers and their corresponding detection methods for major edible oils is presented in Table 2.

## 4. Critical Evaluation of Phytosterol-Based Authentication

### 4.1. Applicability of Phytosterol Markers: When Do Sterols Work as Stand-Alone Markers?

Phytosterols can function as effective stand-alone authenticity markers when the target oil has distinctive sterol features that are absent or present only at trace levels in likely adulterants, when the within-class natural variability associated with cultivar, geography, and season is smaller than the adulteration-induced shift, and when processing and storage do not substantially distort the diagnostic sterol signal [3,19]. Under these conditions, targeted quantification of one or a few sterols or sterol ratios can provide a practical and interpretable screening and confirmation strategy.

This situation is illustrated by olive oil, where changes in campesterol, stigmasterol, and Δ7-stigmastenol have been used to detect adulteration with soybean, corn, and sunflower oils [35,37]. In camellia seed oil, β-amyrin and lanosterol have been proposed as characteristic markers because they differ markedly from those of most common vegetable oils [5,49].

In many real-world situations, however, single markers become insufficient because sterol profiles overlap across botanically related oils, or because refining and storage reshape sterol distributions in ways that mimic or mask adulteration. In such cases, authentication should rely on multi-sterol compositional patterns rather than absolute values of individual compounds. This profile-level strategy becomes especially important when suspected adulterants share similar compositional features, or when the adulteration level is low and must be distinguished from natural variability. For example, olive oil adulteration with hazelnut oil is more difficult than adulteration with common seed oils because the compositional contrast is narrower, even though lupeol-related markers can improve detectability [44]. A similar issue arises in camellia seed oil authentication against olive oil and other high-oleic oils, where bulk compositional similarity reduces the discriminating power of single markers [50]. Moreover, sterols do not always provide sufficient orthogonal information by themselves; combining sterol information with complementary chemical classes can improve discrimination and robustness, as shown in olive oil studies integrating sterols with fatty acids or phenolics [35,44], and in sesame oil studies combining phytosterols with other compositional indicators [52,62].

**Figure 5:**
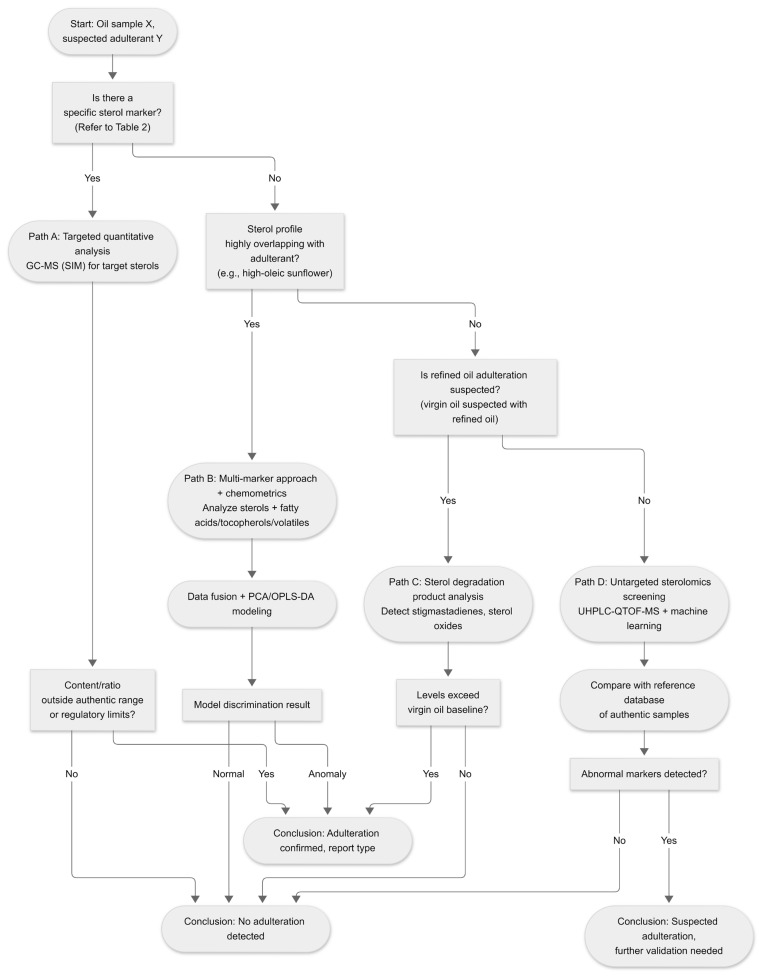
Decision framework for selecting phytosterol-based authentication strategies.

Based on these considerations, Figure 5 presents a decision-oriented framework linking the authentication scenario, expected sources of variability, and the analytical strategy required for method selection and interpretation.

### 4.2. Robustness and Limitations: Confounding Factors and Failure Modes

A major limitation of sterol-based authentication is that sterol composition is not solely determined by botanical origin; it can also be influenced by cultivar, geographical conditions, agronomic practices, and seasonal effects [3,59]. In addition, refining and storage can modify sterol distributions through losses, transformations, or changes in free and esterified forms, potentially shifting profiles in directions that complicate authenticity interpretation [19,64]. Consequently, marker thresholds or typical ranges established in one dataset may not translate directly to new batches, new origins, or different processing states.

This problem is evident in olive oil, where sterol-based evaluation may involve not only discrimination from seed oils but also distinction between virgin and refined materials, for which stigmastadienes become particularly informative [47,48]. Similar concerns apply to camellia seed oil, where discrimination from common vegetable oils is relatively straightforward, whereas differentiation from olive oil or other high-oleic oils is inherently more challenging because the compositional gap is much narrower [5,50].

Another recurrent failure mode is apparent separation that is not truly generalizable. This can occur when datasets are limited in size or diversity, when sample grouping implicitly reflects confounders such as batch, origin, or processing differences, or when the evaluation setting is not sufficiently independent from the model development process. Under such circumstances, reported discrimination performance may be optimistic and may degrade when confronted with new real-world samples. This concern is particularly relevant for chemometric studies that report very high classification performance, because practical robustness depends on whether cultivar, origin, season, processing, and storage variability are adequately represented in the underlying dataset [36,44]. Low-level adulteration also remains scenario-dependent. Relatively low detection limits have been reported for hazelnut oil in olive oil [44] and for rapeseed oil in sesame oil [52,62], but such sensitivity should not be generalized across all oil systems. Therefore, robust sterol-based authentication should be framed as a context-dependent approach: it performs best when sampling captures realistic variability, when marker choices are chemically plausible and scenario-specific, and when conclusions are supported by appropriately independent evaluation and transparent reporting [19,32].

### 4.3. Regulatory Relevance and Standardization Pathways

Current authenticity control is largely framed by standardized methods and compositional limits defined in major regulatory and standard-setting systems, including ISO and national standards (e.g., GB), as well as international frameworks such as EU regulations and Codex Alimentarius. For olive oil in particular, IOC standards have operationalized sterol-related criteria (together with other compositional parameters) as part of routine compliance testing. While these targeted parameters are effective for many conventional fraud scenarios, they are typically based on a restricted sterol panel and fixed thresholds, and can be challenged by context-dependent variability (cultivar, geographical origin, refining, and storage) and by sophisticated adulteration strategies that may partially mimic regulated limits.

In this context, expanded phytosterol profiling and sterolomics are best positioned as a practical extension pathway rather than a replacement of existing regulations. A feasible translation is a tiered scheme in which routine compliance continues to rely on standardized targeted sterol parameters, whereas expanded sterol profiles are reserved for confirmatory testing, dispute resolution, or high-risk cases.

## 5. Challenges and Future Prospects

Despite its analytical power, phytosterol profiling still faces several significant challenges. Firstly, progress is hampered by the lack of comprehensive data on sterol occurrence, as many sterols observed in chromatograms remain unidentified due to a scarcity of authentic reference standards [9]. The creation of extensive, publicly available databases is therefore essential. To be practically feasible, such databases should be built on harmonized analytical protocols and reporting conventions, and should include standardized metadata. Database curation should further incorporate quality-control elements to ensure comparability and traceability across laboratories. Secondly, processing and storage conditions, such as oil refining and thermal treatment, can significantly alter sterol compositional profiles through oxidation and dehydration reactions [67,68]. For example, the formation of β-sitosterol oxides depends on oil type and heating conditions [68], meaning that authentication methods must account for such changes to avoid false-positive results. A further complication arises from natural variability, as the phytosterol compositional profile of crop seeds is influenced by genetics, cultivar, and environmental factors [62,69]. This inherent variation can overlap with the effects of adulteration, complicating data interpretation. Additionally, official analytical methods can be outdated, often missing specific sterols because of derivatization steps or insufficient chromatographic resolution, and thus proving inadequate for detecting sophisticated adulteration practices [70]. Finally, the high-dimensional data produced by modern analytical techniques require sophisticated chemometric tools (e.g., PCA, OPLS-DA) for interpretation [43,51], thereby demanding expertise that often exceeds the standard skill set of routine quality-control laboratories.

Propelled by significant technological and computational advancements, a key development is the paradigm shift from targeted analysis toward non-targeted lipidomics and metabolomics, enabling comprehensive profiling of the sterolome alongside other lipid classes [71]. Llano et al. demonstrated in goldenberries that untargeted metabolomics can identify fatty acyl glycosides that differentiate organic from conventional production systems [72]. Concurrently, the integration of these rich chemical datasets with advanced machine learning algorithms, including Artificial Neural Networks (ANNs), is expected to facilitate the development of more robust and predictive authentication models capable of discerning complex, non-linear patterns and identifying adulteration or oil mixtures that may be difficult to resolve using conventional statistical approaches [36,73]. For routine deployment, AI/ML-assisted models should be supported by rigorous external validation and careful control of common pitfalls, and their transferability across batches, instruments, and laboratories should be explicitly assessed. Improving interpretability and reporting uncertainty will also be important for building confidence in regulatory and industrial decision-making. Furthermore, the push toward high-throughput automation, exemplified by online GC systems and rapid SPE techniques, is expected to make comprehensive sterol analysis faster, more cost-effective, and more accessible for routine monitoring. However, recognizing that no single method is foolproof, the most robust authentication strategies will increasingly adopt a multi-method framework. This approach involves integrating phytosterol profiling with complementary techniques such as DNA-based analysis [57], stable isotope analysis, or spectroscopy (e.g., NMR and NIR), thereby creating a multi-layered database for reliable identification. Finally, interlaboratory validation and proficiency testing are essential to quantify repeatability/reproducibility, establish measurement uncertainty, and define transferable decision limits with controlled false-positive risks, which is a prerequisite for broader standardization and regulatory acceptance.

## 6. Conclusions

Phytosterol profiling has evolved from the analysis of a limited number of sterol markers into a comprehensive strategy for edible oil authentication. While challenges related to variability, processing, and data complexity remain, the ongoing development of extensive databases, advanced analytical platforms, and powerful data analysis tools promises to enhance accuracy and applicability. As the field advances toward non-targeted lipidomics, phytosterol profiling will continue to serve as a cornerstone in safeguarding the integrity and safety of the edible oil industry, as well as in supporting nutritional interventions.

## Figures and Tables

**Table 1:** Comparison of analytical techniques for phytosterol profiling in edible oils.

Analytical Technique	Sample Preparation	Target Phytosterol Forms	Main Advantages	Main Limitations	Typical Applications	References
GC–FID	Saponification; extraction; derivatization	Free phytosterols (main sterols)	Good sensitivity and reproducibility; widely used	Requires derivatization; limited structural specificity	Routine sterol quantification	[12,21,32]
GC-MS/GC–MS (SIM)	Saponification; SPE or similar cleanup; derivatization	Free andminor Sterols; Structural profiling	MS gives structural information; high selectivity	Derivatization needed; limited to volatile forms	Detailed profiling and authentication	[9,33]
Online LC–GC–FID	Automated Saponification & transfer to GC	Free sterols	High throughput; reduced manual steps	High equipment cost; still FID lacks structural information	Routine high-volume sterol analysis	[21]
LC–MS/LC–MS/MS (APCI/ESI)	Minimal cleanup; no derivatization	Free sterols; sterol esters; POPs	Avoids derivatization sensitive & selective	Matrix effect; optimization needed	Comprehensive sterol & oxidation product analysis	[22,23,24]
UHPLC-ESI-QTOF-MS	Optional derivatization; reversed-phase LC	Free & conjugated sterols	High resolution; untargeted potential	Data interpretation complexity	Expanded sterol profiling & detailed compound detection	[25,26,34]

**Table 2:** Characteristic phytosterol markers used for authentication of edible oils.

Oil Type	Adulterant	Phytosterol-Based Marker(s)	Detection Method	Sensitivity	References
Olive oil	Sunflower oil	Increased Δ7-stigmasterol relative to authentic sterol profile	GC-FID/GC-MS	LOD ≥ 2%	[46]
	Soybean oil Corn oil	altered campesterol/stigmasterol ratios	GC-FID	LOD ≥ 4%	[39]
	Rapeseed oil	Brassicasterol appearance	GC/LC	LOD ≥ 5%	[63]
	Refined olive oil/pomace oil	Uvaol; erythrodiol	GC-FID/GC-MS	LOD ≥ 10%	[64]
	Refined vegetable or olive oils	Stigmastadienes (e.g., 3,5-stigmastadiene)	GC-FID/GC-MS	Virgin olive oil: <0.01 mg/kg; refined oils: 0.3–0.9 mg/kg	[50,51]
	Hazelnut Oil	lupeol in total or only in esterified forms of 4,4′-dimethylsterols	GC-MS	LOD ≥ 2%	[44]
Camellia seed oil	Common vegetable oils	β-amyrin and lanosterol	GC-FID/GC-MS	LOD ≥ 30%	[5,52]
	Virgin olive oil	β-Sitosterol (14.1–30.2 mg/100 g)	GC-MS	virgin olive oil (94.3–173.2 mg/100 g)	[53]
	Multiple adulterants	Fluorescent phytosterols & flavonoids	3D fluorescence spectroscopy (EEM)	The correct discrimination rate is 97.78%	[54]
Sesame oil	Rapeseed oil	Brassicasterol appearance	GC-FID	LOD ≥ 5%	[38,65]
	Cottonseed oil	sitosterol, campesterol, and stigmasterol	GC-MS	LOD ≥ 10%	[55]
Avocado oil	Common vegetable oils	β-sitosterol, Δ5-avenasterol	^1^H NMR spectroscopy	LOD ≥ 5%	[66]

## Data Availability

No new data were created or analyzed in this study.
